# Integrative Network Analysis of Predicted miRNA-Targets Regulating Expression of Immune Response Genes in Bovine Coronavirus Infection

**DOI:** 10.3389/fgene.2020.584392

**Published:** 2020-09-30

**Authors:** Olanrewaju B. Morenikeji, Madeleine Wallace, Ellis Strutton, Kahleel Bernard, Elaine Yip, Bolaji N. Thomas

**Affiliations:** ^1^Department of Biology, Hamilton College, Clinton, NY, United States; ^2^Department of Biomedical Sciences, College of Health Sciences and Technology, Rochester Institute of Technology, Rochester, NY, United States

**Keywords:** miRNA, bovine, coronavirus, prediction, gene regulation, immune response

## Abstract

Bovine coronavirus (BCoV) infection that causes disease outbreaks among farm animals, resulting in significant economic losses particularly in the cattle industry, has the potential to become zoonotic. miRNAs, which are short non-coding segments of RNA that inhibits the expression of their target genes, have been identified as potential biomarkers and drug targets, though this potential in BCoV remains largely unknown. We hypothesize that certain miRNAs could simultaneously target multiple genes, are significantly conserved across many species, thereby demonstrating the potential to serve as diagnostic or therapeutic tools for bovine coronavirus infection. To this end, we utilized different existing and publicly available computational tools to conduct system analysis predicting important miRNAs that could affect BCoV pathogenesis. Eleven genes including CEBPD, IRF1, TLR9, SRC, and RHOA, significantly indicated in immune-related pathways, were identified to be associated with BCoV, and implicated in other coronaviruses. Of the 70 miRNAs predicted to target the identified genes, four concomitant miRNAs (bta-miR-11975, bta-miR-11976, bta-miR-22-3p, and bta-miR-2325c) were found. Examining the gene interaction network suggests IL-6, IRF1, and TP53 as key drivers. Phylogenetic analysis revealed that miR-22 was completely conserved across all 14 species it was searched against, suggesting a shared and important functional role. Functional annotation and associated pathways of target genes, such as positive regulation of cytokine production, IL-6 signaling pathway, and regulation of leukocyte differentiation, indicate the miRNAs are major participants in multiple aspects of both innate and adaptive immune response. Examination of variants evinced a potentially deleterious SNP in bta-miR-22-3p and an advantageous SNP in bta-miR-2325c. Conclusively, this study provides new insight into miRNAs regulating genes responding to BCoV infection, with bta-miR-22-3p particularly indicated as a potential drug target or diagnostic marker for bovine coronavirus.

## Introduction

Coronaviruses are pathogenic RNA viruses of the subfamily *Coronovirinae*, engendering severe disease in many bird and mammalian species, such diseases becoming fatal in chickens, pigs, cattle, dogs, and mice (Fehr and Perlman, 2015; Amer, 2018; Ellis, 2019). They are responsible for the ongoing COVID-19 pandemic and the attendant significant morbidity and mortality (Gussow et al., 2020; Lucas et al., 2020). Bovine coronavirus (BCoV) is endemic worldwide and affects both wild and domesticated cattle (Saif, 2010), causing disease affecting the respiratory system and gastrointestinal tract, with pathogenesis ranging from mild to fatal (Underwood et al., 2015; Oma et al., 2016). BCoV has high mortality rates in calves, inducing wasting and flattening of the villi in the GI and a substantial cause of calf diarrhea. It is indicated in winter dysentery, and drives the consequent decrease in milk production in adult cattle. It results in significant economic losses in both the dairy and beef industries, and an outbreak in meatpacking industries can alter the supply chain and meat distribution system of any country (Amer, 2018). To date, there is no effective vaccine for BCoV, which is of significant concern, since the virus has been demonstrated to show low fidelity to its host, infecting other animal in the process (Fehr and Perlman, 2018; Saif and Jung, 2020). Due to the paucity of information on bovine coronaviruses, the possible consequences arising from the current pandemic and its potential to become zoonotic, more research is needed to understand and disentangle host-virus interaction in the context of immune response and disease pathogenesis.

Bovine coronavirus is transmitted via the respiratory or oral-fecal route, and it has been documented that recurrent nasal shedding occurs in infected cattle. Infection is detected using fecal or nasal swabs analyzed via real-time reverse transcriptase-polymerase chain reactions (RT-PCR) that detect nucleocapsid (N) or transmembrane (M) protein-coding genes of the virus (Takiuchi et al., 2006; Decaro et al., 2008; Oma et al., 2016). BCoV has a highly glycosylated spike (S) protein as well as a hemagglutinin esterase (HE), both major participants in the attachment of the virus to the host cell (Saif and Jung, 2020). Viral entry induces activation of both signaling and inflammatory receptors that nurture differential expression of genes, mediating host immune response in the process. Due to the close evolutionary relationship between BCoV and severe acute respiratory syndrome (SARS-CoV), there is a potential for overlap in immune response pathway that benefit our current efforts. It is expected that bovine coronavirus will instigate the response of particular immune-related genes in cattle, with toll-like receptor 9 (TLR9), interleukin-6 (IL-6), and IL-8 implicated in this process (Breuer et al., 2012). Significantly, these genes in the presence of exogenous stimuli, have been demonstrated to mediate the inflammatory response and initiation of innate immunity, regulation of the MAPK cascade, and regulation of T cell proliferation, a major requirement for adaptive and long-lived immune response (Breuer et al., 2012). MicroRNAs have been proposed as players in the regulation process and now occupy a central role in the host immune response to BCoV.

MicroRNAs (miRNAs) are small non-coding regions of RNA which act as post-transcriptional regulatory factors and lessen the expression of their target genes, by either degrading the target mRNAs or inhibit their translation (Lu and Rothenberg, 2018). Regions of their sequences are complementary to that of their target mRNAs, and only 7−8 base-pairs are adequate for target-binding (Mohr and Mott, 2015). A single miRNA can have many target genes, and miRNAs often influence the expression of numerous genes within a functioning pathway (Simonson and Das, 2015; Lu and Rothenberg, 2018). It is thought that more than half of protein-coding mammalian genes are regulated by miRNAs. Bovine miRNAs such as miR-125b and miR-141 have been found to have effects on lactation, miR-122 regulates cholesterol in the liver, and miR-133 controls proliferation of skeletal muscle (Krützfeldt et al., 2005; Chen et al., 2006; Li et al., 2012). In a disease system, the expression of miRNAs can be reduced or amplified (Simonson and Das, 2015), with bovine miRNAs especially found to be differentially expressed upon viral infection (Glazov et al., 2009). miRNAs have shown potential as diagnostic markers, and have been used to distinguish between types of cancer (Mishra et al., 2016), and have been identified as potential biomarkers and promising therapeutic agents (Lu and Rothenberg, 2018). Such therapeutic potential is due to their dynamic ability to target multiple proteins and pathways (Simonson and Das, 2015). They have been studied as a treatment for atherosclerosis, hepatitis C virus, and cancers (Scheel et al., 2017). Additionally, miRNA have been demonstrated to have significant implications in bovine disease elucidation, including immune response to viral infection (Vidigal and Ventura, 2015; Scheel et al., 2017; Morenikeji et al., 2019).

Numerous existing algorithms have proven useful as tools for predicting miRNA targets using seed matches or other compensating factors (Griffiths-Jones et al., 2008; Agarwal et al., 2015; Fan et al., 2016; Hanif et al., 2018). Databases are available to compile associations and interactions between proteins for purposes such as identifying disease genes or biomarkers (Hayashida and Akutsu, 2016; Khurana et al., 2017). Software to assemble functional annotation and significantly associated pathways for genes enhances our understanding of their biological processes (Gene Ontology Consortium, 2004; Mi et al., 2019). Computational tools for predicting the effects of variants have been used to recognize the need for individual preventative treatments and to determine which variants play a role in disease susceptibility (Weil and Chen, 2011; Saint Pierre and Génin, 2014; McLaren et al., 2016).

In this study, miRNAs are predicted to play a significant regulatory role relating to immune response to BCoV infection. Notably, we found no record concerning miRNAs as regulatory factors in bovine coronavirus infection. We utilized a combination of different bioinformatic software to identify miRNAs that target BCoV-associated genes, their functional roles, and existing variants. Increased understanding of the regulatory function of miRNA in relation to BCoV could be valuable for gene therapy and drug development. This study aims to elucidate the role of miRNAs in the host immune response to BCoV, demonstrate miRNA-target interactions, identify potential markers for disease diagnosis, and possible therapeutic drug targets.

## Materials and Methods

### Determination of Genes Associated With Bovine Coronavirus Infection

**FIGURE 1:**
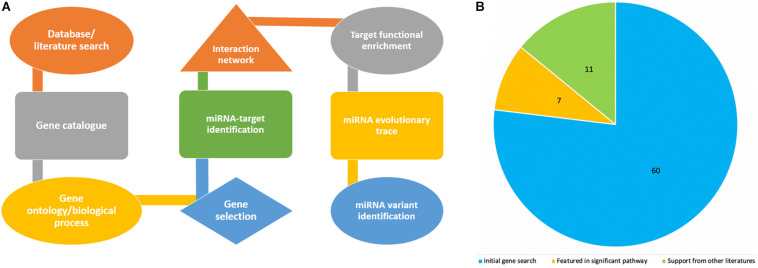
Flowchart showing steps in the project design and analyses process **(A)**, while the pie chart indicates how the number of genes associated with bovine coronavirus were sorted according to significance. **(B)** The blue represents genes that were only from the article by Aich et al. (2007). The orange are genes from the article that also appeared in significant pathways from GO. The green are genes from the article, from significant pathways, and found in supporting literature. The green also includes the gene NANS that was added.

Figure 1A shows the pipeline we utilized for this study, including a step by step flowchart starting from literature search to miRNA variant identification, as previously described (Morenikeji et al., 2019). To begin, we searched for recently published, publicly available literature, pertaining to gene expression, and particularly bovine coronavirus from biomedical science repository PubMed and Google Scholar. Using “BCoV” as the keyword returns 166 results, out of which we identified 77 genes as significant and were included for further analysis (Aich et al., 2007). These 77 genes were used as seed to query Gene Ontology database^1^ with the “*Bos taurus*” and “biological process” as reference. The biological pathways associated with inputted genes and published papers were retrieved. The results were sorted by fold enrichment above 70 and *p*-Values less than 0.005 which eventually catalog a total of 31 significant pathways for further analysis. In all, seventeen genes were reported in these pathways and cataloged for literature search to determine which of these genes have been reported in relation to the greatest number of other coronavirus species (murine, avian, SARS-CoV, feline, porcine, and canine were searched for in particular). Seven genes returned zero supporting articles and were removed from further analysis. The gene NANS (N-acethylneuraminate synthase) was included in the final list of 11 genes due to its relation to the receptor for bovine coronavirus. The eleven genes were then used as seed for further computational analyses.

### Search for miRNAs That Target Identified Significant Genes

Using NCBI^2^, the complete sequences were collected for the 11 genes determined to be significant in bovine coronavirus infection. In order to predict which miRNAs could target the 11 genes, multiple databases were searched for each gene. miRBase (Kozomara and Griffiths-Jones, 2014; Kozomara et al., 2019) was searched using the mRNA sequence of a gene with the “*Bos taurus*” as reference and an *E*-value cutoff of 12. Also, miRNet (Fan et al., 2016) was searched with *Bos taurus* as the reference species, using the “Official gene symbol,” and “targeted by: miRNA.” Additionally, TargetScanHuman (Agarwal et al., 2015) was searched using the gene symbol, and only the “highly conserved” resulting miRNAs were recorded. The returned miRNAs from the databases miRBase, miRNet, and TargetScanHuman were compiled for a total of 69 miRNAs. To determine which miRNAs could target multiple of the 11 genes, an online Venn diagram creator^3^, which determines overlapping elements in the entered lists and creates Venn diagrams to represent them was utilized.

### Creation of Gene−Gene Interaction and miRNA Network

Using STRING^4^, a network for the final 11 genes was created. STRING is a database containing both experimentally determined and predicted interactions between proteins. It creates a visual representation of these interactions. In order to tie the identified miRNAs, target genes and pathways in to a critical network and infer significant biological process/mechanism for gene regulation, Cytoscape version 3.7.2 was utilized to create a network that incorporated the miRNAs, their target genes, and the pathways that the target genes are associated with as described (Oliveira et al., 2018; Lucas et al., 2020).

### Evolutionary Analysis of Concomitant miRNAs Across Gene Set

Three miRNAs that were identified in the previous analysis to have overlapping and multiple targets among the 11 genes included miRNAs bta-22-3p, bta-miR-2325c, and bta-miR-11976 were regarded most significant. The sequences of these concomitant miRNAs were retrieved from multiple sources including miRBase and GenBank. To conduct evolutionary trace analysis, sequences for the concomitant miRNAs from other species were retrieved using Ensembl BLAST^5^. Sequences were retrieved from fifteen species, including gorilla, chimpanzee, human, pig, wild yak, horse, cow, goat, sheep, cat, dog, chicken, mouse, rat, and bat (Table 3). For bta-miR-2325c and bta-miR-11976, the longer NCBI sequence was necessary to return results. One sequence from each species returned was used. Only the region of the returned sequence that aligned with the input sequence, and brief flanking regions, were included for the creation of the phylogeny. MEGA version X was used to align the sequences using MUSCLE alignment for the individual miRNAs and all of them combined (Kumar et al., 2018; Stecher et al., 2020). For the individual miRNAs, a “neighbor-joining” tree was created using the bootstrap method in MEGA version X. A tree containing all three miRNAs was created using iTOL^6^. The display mode was set to “circular.” Phylogenetic analysis was performed in order to determine evolutionary conservation of miRNAs bta-22-3p, bta-miR-2325c, and bta-miR-11976 across species.

### Functional Enrichment of Identified Gene Targets by Concomitant miRNAs

Enrichment analysis was performed using Gene Ontology’s PANTHER tool for the seven genes that were targeted by concomitant miRNAs. These genes were CEBPB, SRC, TLR9, CEBPD, IRF1, NANS, and RHOA. They were searched under “*Bos taurus*” and “biological process.” The results were sorted by the *p*-Values and FDR as described (Oliveira et al., 2018). The significant pathways that related to immune response, and the genes indicated in each, were recorded. For more specific pathways, GeneAnalytics^7^ was utilized. As the species cow is not supported, the seven genes were searched under “human symbols.” The immune-related pathways, their score, and the genes involved in the pathway were recorded. Only the pathways of “high” quality according to GeneAnalytics were considered. miRBase, GenBank UniProt, miRCarta, miRGator, Ensembl, and NON-CODE were searched for functional annotation for the concomitant miRNAs.

### Ascertainment of Variants Within Concomitant miRNAs

The sequences of the four concomitant miRNAs were searched against the cow genome via Ensembl BLAST. The variant feature of Ensembl was used in order to examine the occurrence of variants as they could affect the functionality of the miRNAs. The flanking regions were not considered. For each variant returned, the SNP ID, class, most severe consequence, change tolerance, and location was recorded. The change tolerance score is the Genomic Evolutionary Rate Profiling (GERP) score. The definition of a GERP score is “the reduction in the number of substitutions in the multi-species sequence alignment compared to the neutral expectation” (Huber et al., 2020). GERP scores reflect “rejected substitutions” and quantify negative selection experienced at a particular location (Cooper et al., 2005; Pollard et al., 2010). Previously, and in this study, a cutoff of an absolute value of greater than or equal to 2 was used to determine important variants (Jha et al., 2015).

## Results

### Identification of Significant Genes in BCoV Infection

The article by Aich et al. (2007) was used as the initial source from which to catalog the differentially expressed genes in bovine coronavirus infection (Supplementary Table 1). Since literature pertaining to gene expression in BCoV is limited, further stipulations were placed on the 77 seed genes to ascertain if the gene was significant in the disease. These included association with significant immune-related pathways and supporting literature from other coronaviruses (Table 1). The genes TLR9, RELA and TLR7 appeared in 11, 9, and 8 out of the 31 returned pathways respectively. Interestingly, TLR7 featured in the greatest number of sources pertaining to other coronaviruses. It has been indicated in porcine, avian, human, murine, and feline coronavirus infections (Calzada-Nova et al., 2010; Kameka et al., 2014; Li et al., 2016; Athmer et al., 2018; Malbon et al., 2019). A total of 11 genes comprised the final list: TLR7, TLR9, IRF1, IL-6, CEBPD, TRAF2, RHOA, TP53, CEBPB, SRC, and NANS (Table 1). Figure 1B summarizes the diminution in the number of genes considered for further analysis.

**TABLE 1:** Lists of the genes indicated to be significant in bovine coronavirus infection.

**List of genes**	**Supported by other literatures**	**Final gene list**
TLR7	TLR7	TLR7
TLR9	TLR9	TLR9
IRF1	IRF1	IRF1
IL-6	IL-6	IL-6
CEBPD	CEBPD	CEBPD
TRAF2	TRAF2	TRAF2
RHOA	RHOA	RHOA
TP53	TP53	TP53
RIPK2	CEBPB	CEBPB
SRC	SRC	SRC
CD47		NANS
SRF		
ABCA1		
RELA		
ITGB5		
LCP1		

### Discovery of miRNAs That Target Significant Genes

**FIGURE 2:**
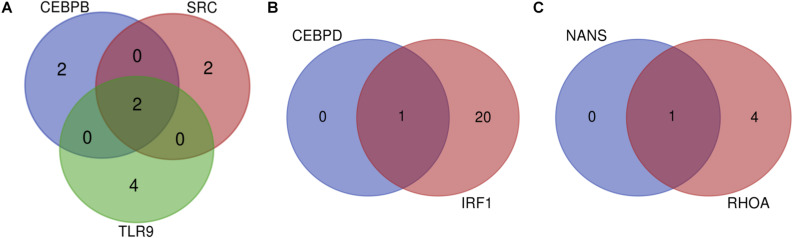
**(A)** Venn diagram to show that CEBPB, SRC, and TLR9 can all be targeted by the miRNAs bta-miR-11976 and bta-miR-11975; **(B)** show that bta-miR-22-3p can target both CEBPD and IRF1; **(C)** showing that NANS and RHOA are both targeted by bta-miR-2325c.

Many miRNAs were predicted for the list of 11 genes as depicted in Table 2. The gene predicted to be targeted by the greatest number of miRNAs was IRF1, which is targeted by 21 miRNAs such as bta-miR-240, bta-miR-22-3p, bta-miR-214, and others. It was followed by TP53 which had 14 predicted miRNAs. Of all the genes, NANS and CEBPD are targeted by the fewest miRNAs with only one predicted miRNA for each. In order to determine the significant miRNAs that could potentially serve as drug targets, concomitant miRNAs were evaluated, with the assumption that miRNAs targeting multiple genes would be choice therapeutic candidates. Both bta-miR-11976 and bta-miR-11975 were predicted to target the genes CEBPB, SRC, and TLR9 (Figure 2A). The genes CEBPD and IRF1 are both targeted by bta-miR-22-3p (Figure 2B) while bta-miR-2325c was predicted to target NANS and RHOA (Figure 2C). These concomitant miRNAs (bta-miR-11976, bta-miR-11975, bta-miR-22-3p, and bta-miR-2325c) were used for further analysis.

**TABLE 2:** List of miRNAs predicted by miRBase, miRNet, or TargetScanHuman for the 11 significant genes.

**Target gene**	**List of miRNAs**
**TLR7**	bta-miR-6528, bta-miR-2405
**TLR9**	bta-miR-2431-3p, bta-miR-2309, bta-miR-11976, bta-miR-11975, bta-mir-2305, bta-mir-2888
**IRF1**	bta-miR-12048, bta-miR-10172-3p, bta-miR-10180-3p, bta-miR-214, bta-miR-2370-3p,
**IL-6**	bta-miR-17-3p, bta-miR-2322-5p, bta-miR-2379, bta-mir-15b, bta-mir-155, bta-mir-16a, bta-mir-223, bta-mir-199a-5p, bta-mir-345-5p, bta-mir-370, bta-mir-504, bta-mir-760-5p, bta-mir-874, bta-mir-2328-5p, bta-mir-2374, bta-mir-1584-5p, bta-mir-2412, bta-mir-2422, bta-mir-2430, bta-mir-2454-5p, bta-mir-2466-3p, bta-mir-2467-5p, bta-miR-22-3p
**CEBPD**	bta-miR-22-3p
**TRAF2**	bta-miR-1584-3p, bta-miR-11981, bta-miR-210
**RHOA**	bta-miR-2325c, bta-miR-2393, bta-mir-2455, bta-miR-133b, bta-miR-133a
**TP53**	bta-miR-19a, bta-miR-19b, bta-mir-671, bta-mir-2438, bta-mir-2442, bta-mir-669, bta-let-7f, bta-let-7c, bta-let-7g, bta-miR-98, bta-let-7b, bta-let-7i, bta-let-7a-5p, bta-let-7d
**CEBPB**	bta-miR-11975, bta-miR-11976, bta-miR-11972, bta-miR-2438
**SRC**	bta-miR-11976, bta-miR-11975, bta-miR-326, bta-miR-9-5p
**NANS**	bta-miR-2325c

### Network Analysis of Genes Significant in BCoV

**FIGURE 3:**
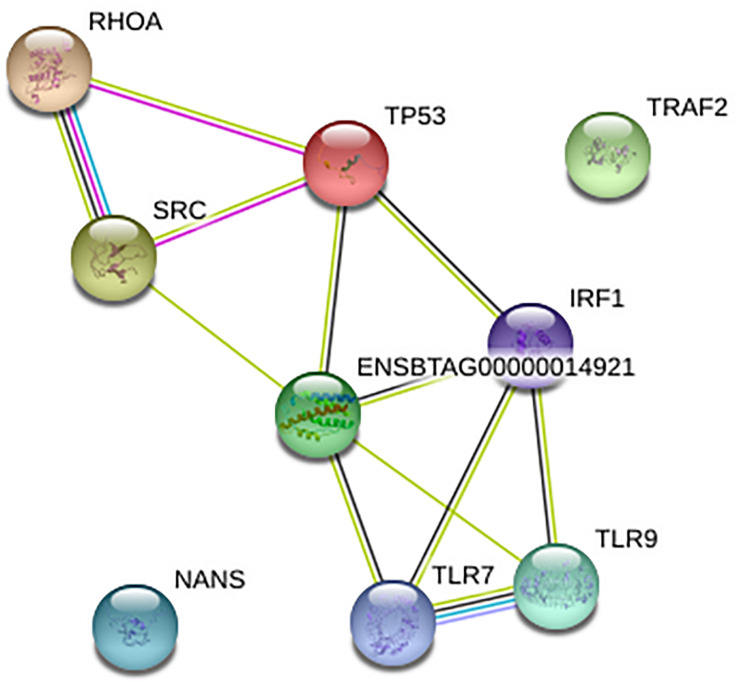
Network for nine of the final genes created using STRING. The Ensembl ID represents cytokine IL-6.

To understand the gene-gene interaction or miRNA-target association that could guide the selection of possible therapeutic agents or discovery of biomarkers for disease diagnosis, we examined the gene relationships in BCoV infection. The online application STRING version 11.0, was used to create a network for the 11 final genes as shown; two of the genes, CEBPB and CEBPD were not found giving a total of nine genes in the network. Of the nine genes that were found in STRING network, only TRAF2 and NANS were not connected with others in the network (Figure 3). IL-6, IRF1, and TP53 are depicted as key drivers of the network. IL-6 is connected to five other genes including TP53, SRC, IRF1, TLR9, and TLR7. IRF1 and TP53 are both connected to four other genes. Many of the interactions, represented in light green, were determined via text-mining. The black lines represent co-occurrence. Two of the interactions come from curated databases, and three of them have been experimentally determined. Two of the interactions have been identified from four different tools. These interactions are between RHOA and SRC and between TLR9 and TLR7.

**FIGURE 4:**
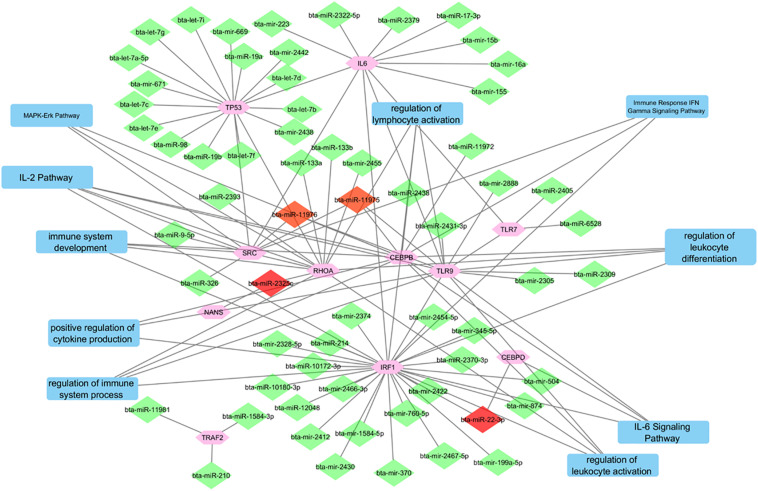
A network incorporating the identified genes, the miRNAs that target them, the interactions between them, and the pathways that they are indicated in.

Figure 4 depicts the interactions between all of the predicted miRNAs, the 11 final genes, the interactions between the genes and the pathways that the genes are associated with. The diamonds represent the miRNAs. miRNAs that were only predicted for one gene appear in green while those predicted to target two or three genes appear in red. Pathways are represented by blue rectangles and other genes are shown in pink. The genes CEBPB, TLR9, IRF1, and RHOA appear as major hubs in the network having several interactions with other elements in the network, and likely having more influence. The genes TLR7, NANS, and CEBPD are connected to the network by only a few interactions. The genes IL-6 and TRAF2 are isolated with connections only to predicted non-concomitant miRNAs.

### Elucidating Evolutionary Relationships of Concomitant miRNAs Across Species

**FIGURE 5:**
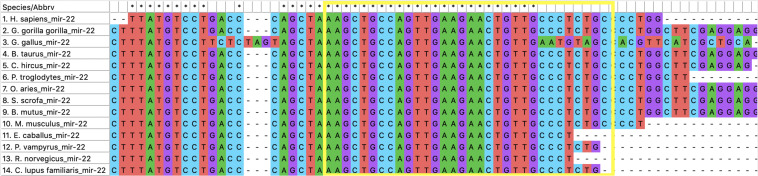
The alignment of miR-22. The yellow box encompasses the sequence of miR-22 for *Bos taurus*.

**FIGURE 6:**
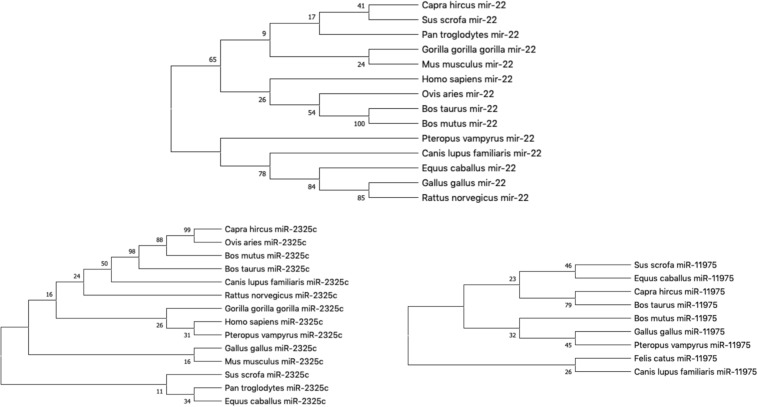
The phylogeny of individual miRNA; **(A)** miRNA-22; **(B)** miR-2325c; **(C)** miR-11975 (Fewer species returned results for miR-11975 than compared to miR-22 and miR-2325c).

Our hypothesis is that miRNAs with lower rates of evolution are conserved across many species and might be candidates to study immune gene regulation in BCoV. Therefore, we performed an evolutionary analysis of the selected candidates from our previous analysis. Information pertaining to the sequences that were used to create the phylogenies for bta-miR-22-3p, bta-miR-2325c, and bta-miR-11975 is shown in Table 3. For bta-miR-11976, no results were found for any of the species searched and was removed from further analysis while miRNA-22 was found in 14 species. The alignment analysis revealed that miR-22 was perfectly conserved across all species that it appeared in Figure 5. From the phylogenetic tree in Figure 6A, it is interesting to note that *Capra hircus* and *Sus scrofa* are closely related. The other ruminants, *Ovis aries*, *Bos taurus*, *and Bos mutus*, are clustered together with *Ovis aries* as the outgroup. While *Capra hircus* and *Sus scrofa* are not closely related species, their miR-22s are indicated to be. Surprisingly, we found *Rattus norvegicus* to be part of the same clade as *Gallus gallus* rather than *Mus musculus*. Lastly, *Gorilla gorilla gorilla and Homo sapien*s most recent common ancestor is at the node with a bootstrap value of 67 and are not sister taxa, as expected.

**TABLE 3:** Characteristics of sequences used to create the phylogenies for bta-miR-22-3p, bta-miR-2325c, and bta-miR-11975.

**Species**	**Genomic location**	**ID**	***E*-value**
	**bta-miR-22-3p**		

Cat	E1:15130930-15130950	MIR22	0.002
Human	CHR_HSCHR17_1_CTG2:1713915-1713935	MIR22HG	0.028
Gorilla	17:1585733-1585753	MIR22	0.002
Chicken	19:5528035-5528055	gga-mir-22	0.001
Cow	19:22811580-22811600	ENSBTAG00000048952, MIR22	
Goat	19:22609267-22609287	MIR22	0.002
Chimpanzee	17:1708699-1708719	ptr-mir-22	0.002
Sheep	11:22431202-22431222	MIR22	0.002
Pig	12:47913344-47913364	TLCD2, MIR22	0.002
Wild yak	JH880403:2914202-2914222	ENSBMUG00000010268, ENSBMUG00000010273	0.002
Mouse	11:75463772-75463792	Mir22hg, Tlcd2, Mir22	0.008
Horse	11:45699966-45699986	MIR22	0.002
Megabat	GeneScaffold_2866:54314-54334	ENSPVAG00000027661	0.002
Rat	10:62299648-62299668	Mir22	0.002
Dog	9:45852535-45852555	ENSCAFG00000045017, MIR22	0.002

	**bta-miR-2325c**		

Cat	X:106820342-106820364		0.002
Human	17:39842584-39842610	ANKRD55	0.002
Gorilla	5:56112509-56112535	ANKRD55	0.04
Chicken	18:8508079-8508102	ENSGALG00000034157	0.044
Cow	25:26423145-26423210	MVP	5.00E-29
Goat	9:34944038-34944062	UBAP1	0.033
Chimpanzee	25:26332743-26332797		9.00E-12
Sheep	1:63813839-63813867		1.00E-04
Pig	24:26434721-26434760		3.00E-11
Wild yak	JH880455:560365-560420	MVP	6.00E-16
Mouse	17:57156609-57156649	PDE4DIP	1.00E-04
Horse	scaffold_8798:49762-49781		0.083
Megabat	X:106087012-106087036	ENSECAG00000042362	0.026
Rat	5:28491754-28491785	LOC100912373	0.002
Dog	1:147508194-147508218		0.13

	**bta-miR-11975**		

Sheep	20:50496857-50496874	ENSOARG00000002813	2.3
Pig	7:120271123-120271141	BCL11B	0.56
Wild yak	JH880919:421961-421979		0.6
Goat	16:24602973-24603015	MIA3	8.00E-13
Chicken	14:2524603-2524624		0.96
Cow	16:26097147-26097192	MIA3	5.00E-17
Cat	B2:145959723-145959740		2.2
Horse	28:35820609-35820627	CARD10	0.57
Megabat	scaffold_62878:368-392		7
Dog	1:8814583-8814602		0.14

miR-11975 and miR-2325c did not display as much conservation as miR-22. For miR-2325c, 14 species such as sheep, mouse, rat, gorilla, and humans were returned from BLAST search results. It is interesting that *Gorilla gorilla gorilla* is the outgroup for *Homo sapiens* and *Pteropus vampyrus* (Figure 6B). *Pan troglodytes* is multiple nodes away from this grouping, and in the same clade as *Equus caballus*. All four ruminants cluster together, but *Capra hircus and Ovis aries* are sister taxa with *Bos mutus* and *Bos taurus* as the outgroups. Once again, *Rattus norvegicus* and *Mus musculus* are not indicated to be closely related.

miR-11975 was only found in 9 species including pig, horse, goat, cow, wild yak, chicken, megabat, cat, and dog. It is interesting to note that *Bos taurus* and *Bos mutus* are multiple nodes apart, whereas they were indicated to be closely related for miR-22 and miR-2325c (Figure 6C). *Bos mutus* is indicated as the outgroup to *Gallus gallus* and *Pteropus vampyrus*, which is surprising.

**FIGURE 7:**
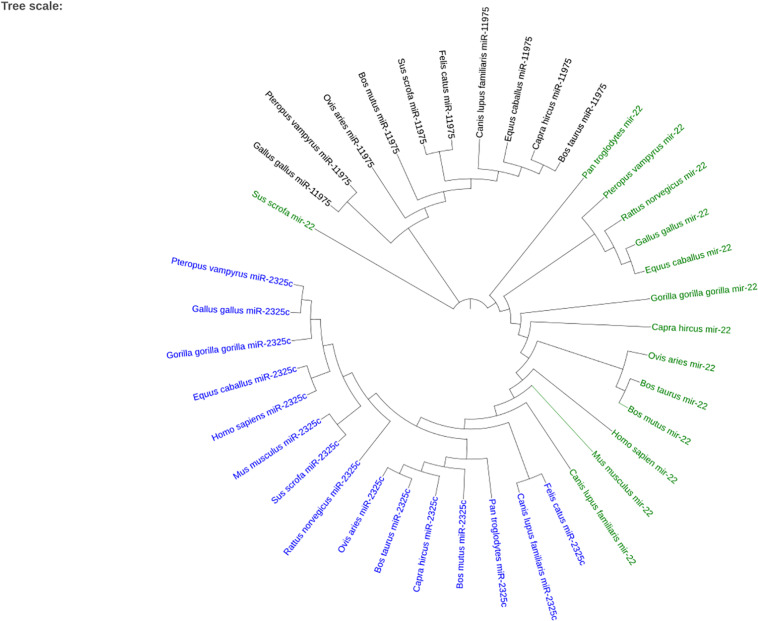
The phylogenetic tree created using iTOL for the three miRNAs that returned homologous sequences from other species on Ensembl. miR-2325c is represented in blue, mir-22 in green, and miR-11975 in black.

When plotting all of the miRNAs together, the three different miRNAs cluster together as seen in Figure 7. *Sus scrofa* miR-22 is the only sequence that is not clustered with other sequences of the same miRNA. It is interesting to note that *Gallus gallus* and *Equus caballus* are sister taxa for miR-22. *Bos taurus* is in the same clade as *Bos mutus* for miR-22 and *Capra hircus* for miR-11975. *Homo sapiens* and *Gorilla gorilla gorilla* are not sister groups in either miR-22 nor miR-2325c. It is especially interesting to note that *Canis lupus familiaris* miR-22 shares a node with branches that terminate in miR-2325c sequences.

### Determining Function of Identified Genes Through Pathways

**FIGURE 8:**
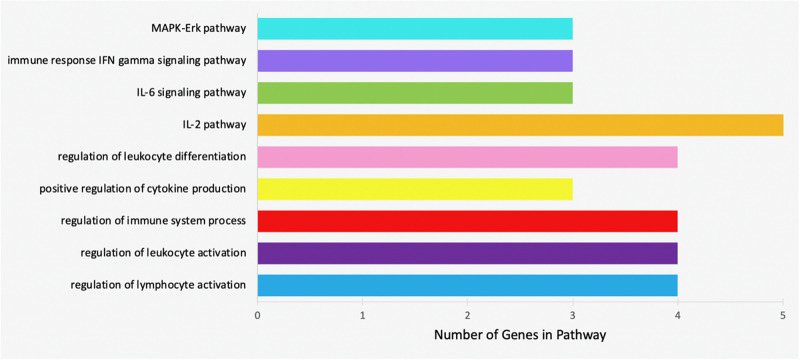
Bar chart representing the number of genes indicated in each of the significant and specific immune-related pathways found using GO and GeneAnalytics.

Specific pathways in which the genes targeted by concomitant miRNAs are indicated in Table 4. Gene Ontology returned the pathways numbered 1-5, while GeneAnalytics returned pathways 6-9. The greatest number of genes, five, was indicated in the “IL-2 pathway” (Figure 8). This pathway includes the genes CEBPB, CEBPD, TLR9, RHOA, and SRC (Table 4). The pathways with the fewest indicated genes, three, were “MAPK-Erk pathway,” “immune response IFN gamma signaling pathway,” “IL-6 signaling pathway,” and “positive regulation of leukocyte differentiation” (Figure 8). Particular combinations of genes appeared in multiple pathways. For example, the genes TLR9, IRF1, CEBPB, and RHOA appeared in four different pathways. bta-miR-22-3p can target CEBPD and IRF1. CEBPD was only present in the IL-6 and IL-2 pathways. IRF1 was indicated in all of the pathways except the “MAPK-Erk pathway” and the “IL-2 pathway.” bta-miR-22-3p will lessen all of the pathways of Table 4. CEBPB, SRC, and TLR9 were predicted to be targeted by bta-miR-11975 and bta-miR. CEBPB was present in all of the pathways including “IL-6 signaling pathway,” “regulation of lymphocyte activation,” and “immune response IFN gamma signaling pathway.” TLR9 was present in six of the pathways such as “regulation of lymphocyte activation” and “positive regulation of cytokine production.” SRC is present in “IL-2 pathway,” “immune response IFN gamma signaling pathway,” and “MAPK-Erk pathway.” As bta-miR-2325c targets RHOA, it will affect the regulation of lymphocyte and leukocyte activation, the regulation of leukocyte differentiation, and the IL-2 pathway. There is considerable overlap in the pathways that will be affected by the concomitant miRNAs. bta-miR-22-3p, bta-miR-11975, and bta-miR-11976 affect all of the pathways in Table 4.

**TABLE 4:** Immune-related and specific pathways in which significant genes are indicated from Gene Ontology and GeneAnalytics.

**S/No**	**Pathway**	**Genes indicated**	***p*-value**
(1)	Regulation of lymphocyte activation	TLR9, IRF1, CEBPB, RHOA	9.11E-06
(2)	Regulation of leukocyte activation	TLR9, IRF1, CEBPB, RHOA	3.09E-09
(3)	Regulation of immune system process	TLR9, IRF1, CEBPB, RHOA	6.29E-07
(4)	Positive regulation of cytokine production	TLR9, IRF1, CEBPB	6.89E-08
(5)	Regulation of leukocyte differentiation	TLR9, IRF1, CEBPB, RHOA	1.11E-08
(6)	IL-2 pathway	CEBPB, CEBPD, TLR9, RHOA, SRC	≥0.001
(7)	IL-6 signaling pathway	IRF1, CEBPB, CEBPD	≥0.001
(8)	Immune response IFN gamma signaling pathway	IRF1, CEBPB, SRC	≥0.001
(9)	MAPK-ERK pathway	CEBPB, RHOA, SRC	≥0.001

### Examination of Variants Within Concomitant miRNAs

Two of the miRNAs, miR-22, and miR-2325c, were found to have polymorphic variants (Table 5). miR-22-3p revealed only one variant, although it could result in a difference in the mature miRNA. The change tolerance for this variant was 3.61. bta-miR-2325c revealed eight variants, all of which could cause intron variance. The variants of bta-miR-2325c had change tolerances ranging from −2.4 to 1.15.

**TABLE 5:** Information pertaining to the variants found for miR-22-3p and miR-2325c when compared to the cow genome.

**miRNA**	**Polymorphism**	**SNP ID**	**Most severe consequence**	**Change tolerance**	**Location**
miR-22-3p	G > A	rs470938322	Mature miRNA variant	3.61	19 22811594
bta-miR-2325c	C > T	rs801070962	Intron variant	–2.4	25 26423208
	C > T	rs480328316	Intron variant	1.15	25 26423196
	G > C	rs460207283	Intron variant	–2.14	25 26423195
	C > T	rs440022857	Intron variant	–2.14	26423194
	C > A	rs724057260	Intron variant	0.14	25 26423163
	C > A	rs721007601	Intron variant	0.07	25 26423160
	A > G	rs385792968	Intron variant	–0.43	25 26423152
	G > A	rs723446659	Intron variant	–0.36	25 26423151

## Discussion

MicroRNAs are short RNA non-coding regions that repress the expression of their target genes post-transcriptionally, and have been implicated in multiple disease conditions, including as potential biomarkers for disease diagnosis and targets for development of therapeutics (Scheel et al., 2017; Lu and Rothenberg, 2018). They have been found to have numerous functions in Bovidae (Krützfeldt et al., 2005). Likewise, the suppression of a such as miRNA-122 has been shown to inhibit human hepatitis C propagation (van Rooij and Kauppinen, 2014) and miRNA-34 aids tumor suppressor in human liver cancer (van Rooij and Kauppinen, 2014; Vidigal and Ventura, 2015). Increasing work is being done to enhance our understanding of their role in bovine immune response to viruses (Lawless et al., 2014; Li et al., 2018; Morenikeji et al., 2019). To the best of our knowledge, the functionality of miRNAs in bovine disease, especially related to bovine coronavirus (BCoV) infection, remains poorly understood. BCoV is an economically costly RNA virus which affects the cattle industry through calf mortality and decreased milk production (Saif, 2010; Amer, 2018). No work has been conducted to determine the role of miRNAs in immune response to BCoV.

In this study, computational tools were employed to predict important miRNAs that target significant immune-related genes indicated in the host response to BCoV. Stringent criteria were placed on the genes whose expression reportedly differed upon infection with BCoV. Conditions included significant association with immune-related pathways and citation in host responses to other coronaviruses. Literature concerning other coronaviruses was considered due to the paucity of published reports regarding gene expression in BCoV. As coronaviruses are genetically similar to each other, it is likely that pathogenesis is similar as well (Siddell et al., 1983; Fehr and Perlman, 2015; McGruder and Leibowitz, 2015; Mousavizadeh and Ghasemi, 2020). The gene NANS, which codes for N-acetyl-9-0-acetylneuraminic acid (van Karnebeek et al., 2016), was included in our study due to its particular involvement in BCoV, despite not satisfying all the stipulations. This sialic acid has been determined to be the receptor onto which the S protein of BCoV binds (Schultze and Herrler, 1993; Belouzard et al., 2012; Park, 2019). Interestingly, NANS is not involved in immune response unlike the remainder of the examined genes. Instead, it only serves as the exclusive entry receptor to BCoV, and therefore not expected to interact with the other genes analyzed in this study. It is also expected that NANS would not appear in literature pertaining to other coronaviruses as the receptor is specific only to BCoV and the human strain hCoV-OC43 (Reguera et al., 2014). bta-miR-2325c was predicted to target NANS, and would therefore repress the expression of the receptor. This miRNA could mitigate viral entry into the host.

This study identified four miRNAs that targets multiple BCoV-associated genes. The concomitant miRNAs were miR-22, miR-2325c, miR-11975, and miR-11976. It is known that an individual miRNA can have multiple phenotypic effects, and by repressing distinct targets, it can be pleiotropic (Valastyan et al., 2009; Chen et al., 2019; Jiang et al., 2019). miRNAs with multiple targets have been shown to have several effects on the pathogenesis of a single disease including human cancer (Ventura et al., 2008; Fazi and Blandino, 2013; Baranwal et al., 2018). Select miRNAs, such as miRNA-155, control diverse functions of the immune system including regulation of inflammatory response, activation of macrophages, and immunological memory, through regulation of numerous immune response genes (Vigorito et al., 2013; Alivernini et al., 2018). In this study, we argue that a concomitant miRNA would have a greater effect on BCoV pathogenesis than a miRNA that targets only a single disease-related gene. These miRNAs could have more than one effect on the host response BCoV. The concomitant miRNAs predicted in this study may serve as effective drug targets as they repress the expression of multiple genes relating to BCoV.

miRNA prediction and functional annotation revealed the potential consequences of gene repression by the concomitant miRNAs. miR-22 targets the genes CEBPD and IRF1. CEBPD is known to enhance inflammatory and immune response genes (Ko et al., 2015). Increased expression of CEBPD also elevates the expression of IL-6 (Hungness et al., 2002). IRF1 mediates both innate and adaptive responses to viral infection such as inducing apoptosis and restricting viral replication (Kanazawa et al., 2004; Kung et al., 2014; Nair et al., 2017; Panda et al., 2019). Both target genes are highly indicated in the immune related pathways found in this study. As these genes partake in the host immune response to the virus, the expression of these genes is beneficial for the host. As miR-22 represses the expression of these advantageous genes, a drug would aim to decrease the expression of miR-22. Previously, miR-22-3p has been identified as a potential drug target and regulator in autoimmune diseases (Wang et al., 2017) and has also been proposed as a biomarker for pancreatic cancer and bovine pulmonary hypertension (Hussein et al., 2017; Grunig et al., 2018). Altering the expression of miR-22-3p has been shown to relieve symptoms of Alzheimer’s Disease, and conserved miRNAs, such as bta-mir-288 and bta-mir-193a, have been identified as drug targets for bovine disease (Ji et al., 2019; Morenikeji et al., 2019). The current study suggests that miR-22-3p may be an effective drug target or diagnostic marker for treating BCoV infection.

Our study also indicated that bta-miR-11976 and bta-miR-11975 target the genes CEBPB, SRC, and TLR9 which are all related to host immune response. Specifically, TLR9 recognizes viral nucleic acids, increases T cell and CD8 + production, induces cytokines, and enhances antibody response (Griebel et al., 2005; Lester and Li, 2014). TLR9 has been found to confer immunity against the respiratory pathogen *Legionella pneumophila* in mice (Bhan et al., 2008). bta-miR-11976 and bta-miR-11975 repress TLR9, and therefore their expression may decrease the host’s capacity to response to a BCoV. A drug repressing bta-miR-11976 and bta-miR-11975 may decrease the severity of BCoV infection. miRNA-2325c has not been functionally annotated nor studied to the best of our knowledge but through the *in silico* tools, we are able to infer the regulatory function of this miRNA. miRNA-2325c targets the genes NANS and RHOA. NANS codes for BCoV’s receptor. As bta-miR-2325c targets RHOA, it will decrease the regulation of lymphocyte and leukocyte activation, decrease the regulation of leukocyte differentiation, and affect the IL-2 pathway. RHOA has been determined to regulate the cell cycle, cytoskeleton formation, and cell morphology (Oishi et al., 2012; Satoh et al., 2012). We are hereby proposing that miRNA-2325c therefore may regulate structural components of bovine cells which might have a protective function against the virus invasion by repression of NANS that codes for BCoV receptor.

Recognition of miRNA targets in conjunction with network analysis creates an extensive visualization of the identified genes’ biological processes. Gene interaction networks have previously been used to identify important genes associated with diseases such as bovine mastitis, trypanosomosis, tuberculosis, and human cancer (Aranday-Cortes et al., 2012; Al-Aamri et al., 2019; Li et al., 2019; Morenikeji et al., 2019). Certain genes were revealed to be central in the gene interaction network of this study. One such gene, IL-6, is responsible for numerous host viral responses. For example, it affects inflammation, activates B cells, controls the differentiation of T cells, and induces antibody synthesis (Tanaka et al., 2014). While none of the concomitant miRNAs target IL-6, the miRNA miR-22-3p would affect the expression of IL-6 through its regulation of CEBPB and IRF1. IL-6 was found to be connected to IRF1 via co-occurrence and text-mining. Functional annotation of CEBPB indicates that it is associated with IL-6 expression (Ko et al., 2015; Delgobo et al., 2019). Both these genes were also found to be associated with the IL-6 signaling pathway. Expression of miR-22-3p would therefore effect more than simply its target genes as it might affect several pathways or biological processes.

Furthermore, evolutionary analysis revealed that miR-22 was perfectly conserved across all of the species it was compared to including humans, sheep, bat, and mouse indicating its functional role has been selected and preserved during evolution. Flanking regions were also perfectly conserved. This evolutionary conservation reveals the fact that mutations were not tolerated in this region, suggesting that miR-22 has an important functional role that is shared across the species it appears in.

Moreover, miR-22-3p was found to have a variant that results in a difference in the mature miRNA. This variant had a change tolerance of 3.61. The change tolerance score, which is the Genomic Evolutionary Rate Profiling (GERP) score, is defined as “the reduction in the number of substitutions in the multi-species sequence alignment compared to the neutral expectation” (Huber et al., 2020). GERP scores reflect “rejected substitutions” and quantify negative selection experienced at a particular location (Cooper et al., 2005; Pollard et al., 2010). Commonly, a cutoff of an absolute value of greater than or equal to 2 is used to determine important variants (Jha et al., 2015). With a GERP score of 3.61, the variant of miR-22-3p experienced 3.61 fewer substitutions at that location compared to the neutral rate of evolution. This high preclusion of substitutions shows that the variant is deleterious. miR-2325c revealed eight variants. Only three of them had a GERP score with an absolute value greater than two. The GERP scores were negative, indicating that substitutions occur at a rate that is greater than neutral, suggesting that substitutions are tolerated in these locations, and it is likely that they are not detrimental. The variants may even be beneficial, as previously described in which similar variation in mature miRNAs targeted CD14 gene, thus regulating its expression during bovine trypanosomosis (Morenikeji et al., 2020). Further work is needed to validate these predictions with *in vitro* analyses examining miRNA expression, binding, and their effect on the host immune response to bovine coronaviruses.

## Conclusion

This study reveals computational evidence that particular miRNAs could regulate the host immune response to bovine coronavirus. Eleven genes were identified as predominant players in the host response, and an integrated pipeline was developed in order to determine the importance of the miRNAs that targeted them. The concomitant miRNAs were assumed to play a larger role in disease pathogenesis, as they could affect the expression of multiple BCoV-associated genes such as TLR9, IRF1, and RHOA. Functional capacities of the predicted miRNAs were inferred using annotations of the target genes, their network interactions, as well as their associated pathways. Some important pathways identified include the IL-6 signaling pathway, regulation of lymphocyte activation, and the IL-2 pathway. A critical function of miR-22-3p is suggested as the miRNA was found to be completely conserved across numerous species. miR-22-3p was also found to contain a potentially deleterious variant. Overall, this study expands our understanding of the host immune response to BCoV. Four miRNAs, bta-miR-11975, bta-miR-11976, bta-miR-22-3p, and bta-miR-2325c that were identified could serve as potential biomarkers with which to diagnose BCoV and as drug targets for therapeutic treatments. However, further work is recommended to validate these predictions with *in vitro* analyses examining miRNA expression, binding, and their effect on the host immune response to BCoV.

## Data Availability Statement

All datasets generated for this study are included in the article/Supplementary Material.

## Author Contributions

OBM and BNT conceptualized and designed the experiments, revised the manuscript, and contributed to the discussion and scientific content. OBM, MW, ES, KB, and EY carried out the experiments, analyzed the data, and drafted the manuscript. All authors read and approved the final version of the manuscript.

## Conflict of Interest

The authors declare that the research was conducted in the absence of any commercial or financial relationships that could be construed as a potential conflict of interest.

## Footnotes

1 http://geneontology.org/

2 https://www.ncbi.nlm.nih.gov/

3 http://bioinformatics.psb.ugent.be/webtools/Venn/

4 https://string-db.org/

5 https://useast.ensembl.org/Multi/Tools/Blast

6 https://itol.embl.de/tree/

7 https://ga.genecards.org/
