# Supplementary material for: Integrative Network Analysis of Predicted miRNA-Targets Regulating Expression of Immune Response Genes in Bovine Coronavirus Infection
Source: Front Genet. 2020 Sep 30;11:584392. doi: 10.3389/fgene.2020.584392 (PMC7554596; doi:10.3389/fgene.2020.584392)
Supplement: Supplementary file 1 [file Table_1.pdf]

Suppl Table 1. List of the initial seed genes (from Aich et al., 2007)

TLR7	RIPK2	HMGA1	C1R
SRC	YWHAB	CXCL12	C1S
TLR9	RPS6KA3	JAK3	C4BPA
p38	SERPING1	p64	CAMK2G
IL-6	SIN3A	CcnE1	CEBPD
IRF3	CEBPB	Cdc2	CSRP2
TIMP1	IRF1	Cdkn1A	GRB10
CDK4	CAMK2D	Cdc20	GSK3A
TYK2	CD81	CdcA8	IFNGR2
TLR8	CD47	GRAP2	ITGB1
CASD1	CCNE1	IL-10RB	ITGB5
PLA2G2D	ISGF3G	IRS2	MAP2K2
FOS	C1QB	LCP1	NCOR1
BoLA	CDKN1A	MAP2K6	NFATC4
JUN	PRDX2	PRDX4	PAK2
YWHAZ	NANS	ABCA1	PRDX6
JUNB	ITGA2B	ARPC3	PRKCG
MEF2B	TP53	ARPC5	RAPGEF1
RHOA	SRF	TRAF2	RELA
